# Evaluation of the new Egyptian undergraduate medical program from students’ perspectives: a nationwide cross-sectional study

**DOI:** 10.1186/s12909-026-10200-2

**Published:** 2026-09-08

**Authors:** Somaya Hosny, Hany Atwa, Mona S. Ghaly, Ahmed M. Makhlouf, Sanaa Radi, Zeinab N. A. Said, Hala Salah, Abdelrahim Shoulah, Asmaa Abdelnasser

**Affiliations:** 1 Faculty of Medicine, Suez Canal University, Ismailia, Egypt; 2 College of Medicine and Health Sciences, Arabian Gulf University, Manama, Kingdom of Bahrain; 3 Faculty of Medicine, Assiut University, Assiut, Egypt; 4 Badr University in Cairo, Cairo, Egypt; 5 Faculty of Medicine, Alexandria University, Alexandria, Egypt; 6 Faculty of Medicine (for Girls), Al-Azhar University, Cairo, Egypt; 7 Faculty of Medicine, Cairo University, Cairo, Egypt; 8 Faculty of Medicine, Banha University, Banha, Egypt; 9 Ibn Sina National College for Medical Studies, Jeddah, Saudi Arabia

**Keywords:** Undergraduate medical program, Students' experiences, Students’ perspectives, Competency-based education, Program evaluation

## Abstract

**Background:**

The global emphasis on medical education reform highlights the need for essential competencies to meet evolving healthcare demands. In Egypt, significant reforms in 2018 introduced a competency-based, integrated undergraduate medical curriculum—a five-year program followed by a two-year internship aligned with the National Academic Reference Standards for Medicine. This study assesses graduates’ perceptions and experiences with the newly implemented reformed national medical program.

**Methods:**

This descriptive, cross-sectional study was conducted between December 2023 and December 2024. Using a convenience sampling, it included 7,264 recent graduates from 27 Egyptian medical schools. Data was collected via an online survey comprising 45 Likert-scale items assessing various aspects of the program. Additionally, three open-ended questions explored graduates’ most and least favorable aspects of the program and improvement suggestions. The survey underwent validity testing through exploratory factor analysis and expert review, as well as reliability testing through Cronbach’s test.

Data analysis included descriptive and inferential statistics, such as independent samples t-tests and one-way ANOVA, with significance set at *p* < 0.05. Effect sizes (Cohen's d and η²) were calculated for all significant group comparisons to assess practical significance. Responses to open-ended questions were categorized and analyzed using quantitative content analysis (thematic categorization with frequency counts). Ethical approval, informed consent, and participant anonymity were ensured.

**Results:**

The study received responses from 7,264 graduates (40.7%). Validity and reliability tests confirmed the survey's robustness, with Cronbach's alpha of 0.98 and exploratory factor analysis identifying four factors, accounting for 64.53% of total variance. Survey responses indicated moderate overall satisfaction across program dimensions, with mean scores ranging from 2.71 to 3.41. Key strengths included integration of basic and clinical sciences, diverse learning opportunities, early clinical exposure, emphasis on critical thinking, self-directed learning, and faculty support. Male students, high achievers, and those with strong English proficiency or university housing reported slightly higher satisfaction, though effect sizes were small. Challenges such as heavy workload, difficulty adapting to the new curriculum, and frequent assessments were noted. Quantitative content analysis of open-ended responses reinforced the quantitative findings, offering additional descriptive insights into students’ experiences.

**Conclusion:**

The shift to Competency-Based Medical Education in Egypt marks a significant reform to better prepare graduates for modern medical practice. Key strengths include subject integration, diverse learning opportunities, early clinical exposure, emphasis on critical thinking and self-directed learning. However, challenges like increased workload, frequent assessments, and difficulties adapting to the new curriculum were noted.

**Supplementary Information:**

The online version contains supplementary material available at https://doi.org/10.1186/s12909-026-10200-2.

## Background

The global call for reform in medical education has been growing steadily. Such reform represents a profound shift in the medical and health professionals’ knowledge, skills, and values. It highlights the importance of equipping medical students with essential competencies, including critical thinking, clinical decision-making, and teamwork, to address the evolving and diverse needs of healthcare [1]. Educational Reform refers to the process of making changes and improvements in the educational system. Several factors have been reported for the necessity of reform in medical education [2]. These factors include the alignment with the rapid advancements in medical science and technology to keep pace with these changes [3], the compliance with requirements of the health system and the population it serves [4–6], and the evolving healthcare landscape which requires medical graduates to have a diverse set of skills, including clinical competence, communication, and teamwork [1]. Additionally, increasing global health challenges, such as pandemics and chronic diseases, emphasize the need for a more integrated and practical approach to medical education [7].

Although evidence of medical practice in Egypt dates back to 2,500 B.C., medical education was incorporated into the university system only in 1919. From that time until the turn of the millennium, there were few national reform initiatives [8].

In June 2016, the planning and development committee of the medical sector, which oversees all medical schools in Egypt under the governance of the Ministry of Higher Education, established a subcommittee called the Reform of Undergraduate Medical Program (RUMP) to create a strategic plan for undergraduate medical education (UME) and implement the necessary changes. In 2017, the Supreme Council of Egyptian Universities (SCU) approved and enforced significant reforms in medical program through primarily changing the 6-year academic and 1-year internship program into 5 years and 2 years, respectively. SCU restructured the medical program timeline to align with international standards, reduce theoretical overload, improve efficiency, and strengthen graduates’ clinical competence through extended supervised training. This reform was preceded by a thorough assessment of the existing situation, an awareness campaign, and a national faculty development program to ensure smooth implementation [9]. The new program focused on competency-based education, integrated learning methods, and the early introduction of clinical exposure. These changes aimed to produce well-rounded graduates who are better equipped to meet the demands of modern healthcare environments. This new medical program was aligned with the 2017 National Academic Reference Standards for Medicine (NARS-Medicine) to guide schools in creating their new integrated curricula. The reform process took into consideration the Egyptian context and the innovative medical education strategies that prepare medical students to practice in the evolving field of medical science [9].

All medical schools in Egypt started the implementation of the new program in September 2018. The Supreme Council of Medical Universities (Medical education sector) assigned a committee to follow up and support the new medical programs in all the Egyptian medical schools. As any reform, this process came with its challenges, thus among this committee objectives were to regularly implement short term remedies for the emerging issues and to conduct a comprehensive program evaluation after the graduation of the first cohort to assess the overall impact.

Program evaluation is a crucial process in higher education, ensuring that curricula remain effective, relevant, and aligned with evolving academic and professional demands. Evaluating an undergraduate medical program requires input from various stakeholders, including faculty, administrators, and, most importantly, students. As the primary recipients of education, students provide invaluable insights into the curriculum’s strengths, weaknesses, and areas for improvement [10]. Their perspectives help educators assess whether the program meets learning objectives, fosters clinical competencies, and adequately prepare graduates for real-world medical practice [2]. Moreover, stakeholder feedback is essential for continuous quality enhancement, allowing institutions to make data-driven modifications to teaching methods, assessment strategies, and resource allocation [11]. Integrating students’ voices into program evaluation not only ensures a student-centered approach but also enhances engagement and satisfaction, ultimately contributing to better learning outcomes [12].

This study evaluates the new Egyptian undergraduate medical program from the perspectives of students, framed as a national program evaluation of the implementation of Competency-Based Medical Education (CBME), examining their satisfaction levels, perceived strengths, challenges encountered, and recommendations for improvement. The findings will contribute to medical education literature and inform policymakers and educators in Egypt and beyond.

## Methods

### Study design

This study employed a descriptive, cross-sectional, survey-based research design to explore the perceptions and experiences of the graduates of the Competency-Based Medical Education (CBME) “5 + 2” medical program. The study was conducted during the period from December 2023 to December 2024.

### Study population and sample characteristics

The study targeted all fresh graduates from the newly implemented medical program across 27 colleges of Medicine in Egypt, where students completed a five-year course of study. A convenient self-selection sampling method was used, where all the graduates were invited to participate. Out of 17,838 medical graduates, 7264 responded to the survey.

### Evaluation framework

We adopted the Kirkpatrick Model as the primary framework, which evaluates educational programs across four levels: reaction, learning, behavior, and results [10]. The survey, used in this study, primarily assessed Level 1 outcomes, focusing on participants’ perceptions and satisfaction with the reformed medical education program.

### Survey

A structured survey form was developed by the researchers after thoroughly reviewing existing studies and validated instruments to assess fresh graduates’ perceptions of the new CBME (5 + 2) medical program. The survey covered five dimensions, including program goals, curriculum content, learning resources, faculty support, and overall experience. It consisted of 45 items, along with an additional item measuring overall satisfaction with the learning experience. Responses were recorded on a 5-point Likert scale (1 = Strongly Disagree to 5 = Strongly Agree).

In addition, the three open-ended questions were appended to the survey. Those questions were: *“What did you like most about the program?”*, *“What did you dislike most about the program?”*, and *“What are your suggestions for improving the program?”*.

Since the survey was researcher-developed, reliability and validity testing were conducted to ensure data credibility. Internal consistency was assessed using Cronbach’s alpha, while content and construct validity were established. Content validity was ensured through expert review by seven independent specialists, including medical educationists, public health consultants, and a psychometrician. Exploratory Factor Analysis (EFA) was performed to confirm construct validity. Prior to distribution, the survey underwent a rigorous pre-administration review process: expert review by seven independent specialists led to item revision and rewording, ensuring clarity, relevance, and content coverage before the instrument was finalized and distributed.

The survey was transformed into an online form (through SurveyMonkey) and distributed electronically via official email addresses, with each college managing distribution to its own graduates. Responses were collected over 12 weeks.

### Data analysis

#### Exploratory Factor Analysis (EFA)

Principal component analysis with Varimax rotation was utilized in the Exploratory Factor Analysis (EFA). The number of factors to extract was determined based on multiple criteria, including the Kaiser criterion (retaining factors with eigenvalues > 1) [13], Bartlett’s test of sphericity [14], the Scree test (identifying the inflection point on the Scree plot) [15] and the cumulative percentage of variance explained [16].

Factor solutions were assessed using established interpretability criteria [17]:

- A valid factor required at least three items with loadings of 0.30 or higher.
- Items within the same factor needed to share a common conceptual meaning.
- Items that loaded onto a different factor were considered to measure a distinct construct.

#### Analysis of responses

The survey data was analyzed using IBM SPSS v.29, applying both descriptive and inferential statistics to evaluate graduates’ perceptions. Overall satisfaction was compared based on gender, nationality, last obtained grade, school type, English language proficiency, and residence during college time. Independent samples t-test and one-way ANOVA were used for comparisons, with statistical significance set at *p* < 0.05.

To quantify the practical magnitude of statistically significant differences, effect sizes were calculated: Cohen’s *d* for independent-samples t-tests (gender and nationality comparisons) and eta-squared (η²) for one-way ANOVA comparisons (grade, school type, English proficiency, and residence), interpreted using conventional benchmarks (Cohen’s *d*: negligible < 0.2; η²: negligible < 0.01, small ≥ 0.01, medium ≥ 0.06, large ≥ 0.14). Analyses involving school type, English language proficiency, and residence during college were based on a subsample of *N* = 5,916, as these items were not completed by all respondents. This is reported transparently in Table 5.

The responses to the open-ended questions were analyzed using quantitative content analysis. Two members of the research team independently categorized all responses into thematic categories; inter-rater agreement was checked and any discrepancies were resolved through discussion and consensus until full agreement was reached. Inter-rater reliability (e.g., Cohen’s κ or percentage agreement) was not calculated at the time of coding, as independent, pre-consensus codes were not retained separately for each rater, which is reported as a limitation. Responses were then counted and results were presented as frequencies and percentages. Representative student quotes were selected to illustrate each category. This approach constitutes quantitative content analysis rather than qualitative research in the interpretive sense, as it was designed to enumerate and summarize response themes rather than to explore their deeper meaning or context.

## Results

This descriptive, cross-sectional, survey-based study aimed to evaluate the new Egyptian CBME (5 + 2) undergraduate medical program from the perspectives of its fresh graduates. Using a detailed, researcher-made survey instrument that assessed various dimensions, responses were collected from a representative sample. A total of 7264 fresh graduates responded to the survey, yielding a response rate of 40.7%.

The results are presented here in two sections, which are: validity and reliability studies of the survey and analysis of survey responses (including quantitative content analysis of the responses to the open-ended questions).

### Section 1: validity and reliability studies of the survey

#### Reliability study

The internal consistency of the survey was evaluated using Cronbach’s alpha, yielding a coefficient of 0.98, indicating excellent reliability. However, this high value may also reflect a degree of item redundancy across the 45-item instrument. Future studies should examine item-total correlations and inter-item correlations to identify potentially redundant items and streamline the instrument accordingly.

#### Content validity

A critical review of the survey led to modifications, including rewording and item additions to enhance clarity and relevance. Notable revisions included:

- Item #22 was changed from *“The learning opportunities provided by the program made me able to conduct a research study”* to *“The learning opportunities provided by the program helped me develop my research skills”* to better reflect the intended meaning.
- Item #23 was modified from *“The learning resources provided by the program helped me pass final exams”* to *“The learning resources provided by the program helped me prepare for assessments”*, making it more inclusive of various evaluation methods.
- Item #47, which assessed students’ perceived readiness for practice (*“I feel ready to graduate and obtain a license to practice the profession”*), was removed. Given that students had not yet completed their internship, it was deemed premature to expect them to determine their readiness for professional practice.

#### Construct validity

**Fig. 1:**
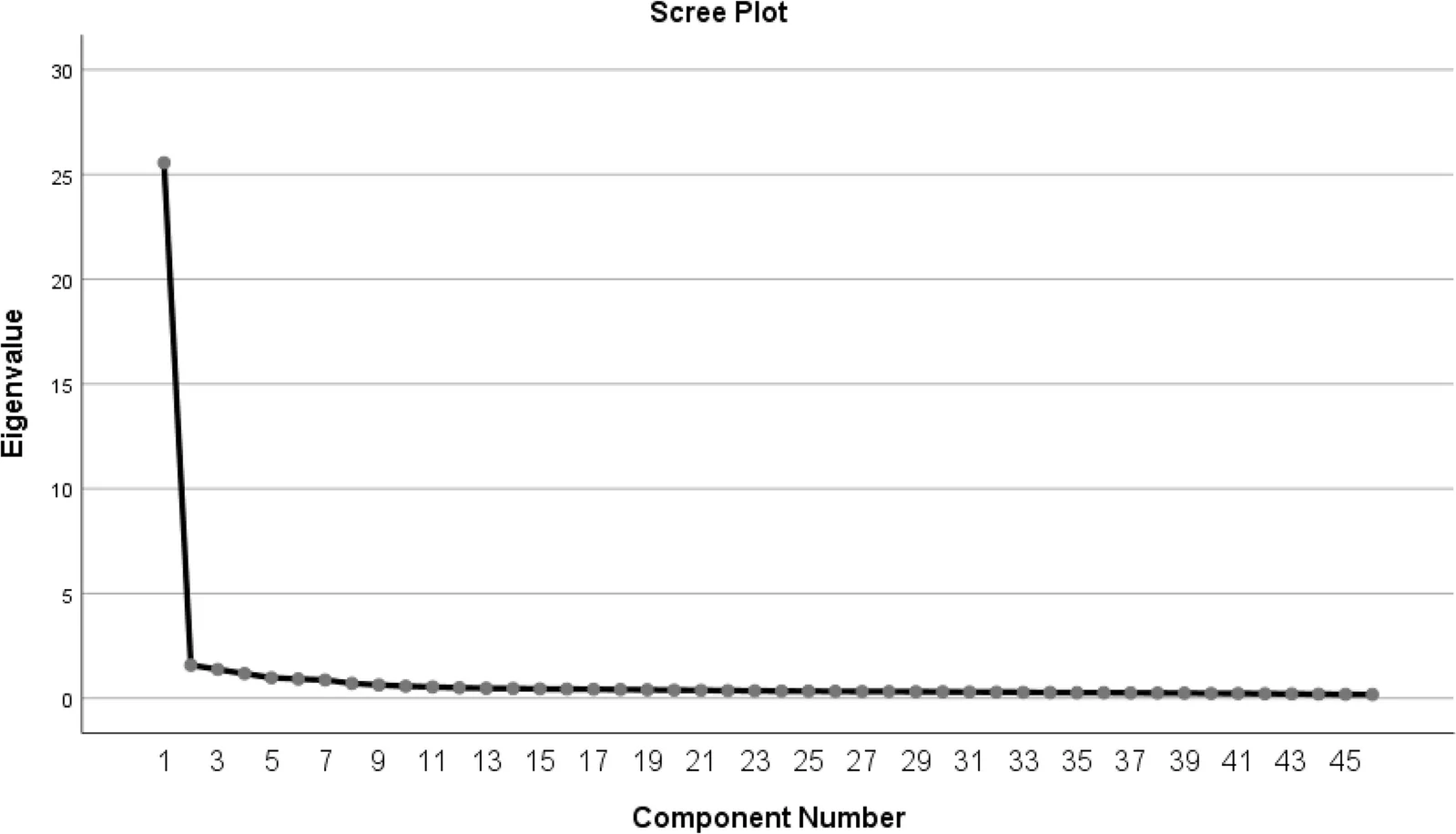
Scree plot of EFA

Construct validity was established through Exploratory Factor Analysis (EFA) (Table 1 and Fig. 1), which yielded the following findings:

- The dataset, comprising 7264 responses, was deemed suitable for factor analysis. The Kaiser-Meyer-Olkin (KMO) Measure of Sampling Adequacy was 0.988, and Bartlett’s Test of Sphericity was significant (χ² (1035, *N*=7264) = 286798.19, *p* < 0.001), confirming the appropriateness of the data.
- All factors contained at least three items, with no cross-loading observed, and all retained items had loadings exceeding 0.30 on their respective factors.
- Factor extraction and rotation identified four factors with Eigenvalues > 1, collectively explaining 64.53% of the total variance.

**Table 1:** Exploratory Factor Analysis (EFA)

Item No.	Item Text	Factor 1	Factor 2	Factor 3	Factor 4
1	The program’s mission and goals were clear, appropriate, publicized, and reflected my aspirations to study in the program and obtain the degree	0.678			
2	The learning outcomes of the program were generally clear	0.682			
3	The learning outcomes of the different courses were clear	0.681			
4	The program achieved the principle of integration between the basic sciences and each other, and between them and the clinical sciences	0.629			
5	Program content was suitable for the requirements of the future doctor	0.670			
6	I was effectively prepared to study in the program as it was a new program	0.659			
7	The learning environment in the program was stimulating and encouraging	0.603			
8	The program contains sufficient and appropriate elective courses to prepare graduates for various fields of specialization	0.535			
9	The program offered enough learning opportunities (i.e., classes, lectures, clinical sessions, community-based activities …)	0.517			
10	Teaching methods were variable and appropriate for the learning outcomes	0.556			
11	The instructors used interactive teaching/learning methods (e.g., small group activities, discussion forums …)	0.412			
12	Assessment methods were enough for measuring the learning outcomes	0.595			
13	Teachers used the agreed-upon assessment methods as mentioned in each course specification	0.512			
14	Assessment methods were fair	0.546			
Eigenvalue: 25.56 | Variance: 55.57% *Factor 1 Label: “Program Learning Outcomes*,* Content*,* Educational Strategies*,* and Student Assessment”*					
15	The instructors provided me with constructive feedback on strengths and areas for improvement whenever possible		0.550		
16	The program offered adequate opportunities to participate in research projects		0.483		
22	The learning opportunities provided by the program helped me develop my research skills		0.429		
24	Physical facilities (i.e., lecture halls, laboratories, small group rooms …) were adequate and suitable		0.434		
25	The student computer labs were sufficient for my needs		0.525		
26	Adequate facilities were available for extracurricular activities (including sporting and recreational activities)		0.642		
27	Field training activities (clinical training, practicum, cooperative training) were effective in developing my clinical skills		0.469		
28	I was made aware of the academic support services provided (e.g., academic advising, specialized services for disabled students, study skills workshops …)		0.667		
29	The program provided me with counseling and academic support whenever I needed it		0.737		
30	The instructors were available for consultation and advice		0.580		
31	The teachers were supportive and responsive to my needs and encouraged me to do my best		0.641		
32	My academic progress was continuously monitored by my teachers who provided me with feedback on my achievement		0.720		
33	The program faculty had significant knowledge of the program content, and this applies to technicians in laboratories, clinics, and training settings		0.605		
34	Academic support services provided by the program were generally satisfactory		0.632		
Eigenvalue: 1.58 | Variance: 3.44% *Factor 2 Label: “Academic Support*,* Learning Resources*,* Research Opportunities*,* and Facilities”*					
35	The program goals inspired me to pursue a career in medicine			0.498	
36	The program helped me develop the knowledge and skills I need to be a successful doctor			0.557	
37	The program helped me to keep up to date with the latest developments in medicine			0.552	
38	The program helped me develop the ability to investigate and solve new problems			0.667	
39	The program helped me develop self-directed learning skills			0.718	
40	The program helped me develop the ability to work effectively in teams			0.703	
41	The program improved my communication and interpersonal skills			0.718	
42	The program helped me develop critical thinking skills			0.717	
43	The program helped me develop professionalism and commitment to ethical practice			0.678	
44	The program helped me develop skills in using technology in learning			0.675	
45	The program helped me develop and improve my abilities in the field of innovation and skill acquisition			0.582	
Eigenvalue: 1.37 | Variance: 2.98% *Factor 3 Label: “Program Impact on Professional Development and Essential Competencies”*					
17	The learning resources (e.g., textbooks, guides, web-based sources …) provided by the program were adequate to meet my needs				0.693
18	The learning resources provided by the program were up to date and accurate				0.672
19	The learning resources provided by the program were accessible and easy to use				0.749
20	The learning resources provided by the program supported students’ different learning styles (visual, auditory, kinesthetic)				0.589
21	The learning resources provided by the program helped me to become an independent learner				0.610
23	The learning resources provided by the program helped me prepare for assessments				0.547
Eigenvalue: 1.17 | Variance: 2.55% *Factor 4 Label: “Quality*,* Accessibility*,* and Effectiveness of Learning Resources”*					

Following EFA, the survey retained all the original 45 items, grouped into the four factors, named based on the highest-loading items and their conceptual relevance:

1. Factor 1 explained 55.57% of the variance, with an eigenvalue of 25.56. Fourteen items loaded onto it, with values ranging from 0.412 to 0.682. This factor was labeled *“Program Learning Outcomes*,* Content*,* Educational Strategies*,* and Student Assessment”*.
2. Factor 2 accounted for 3.44% of the variance, with an eigenvalue of 1.58. Fourteen items loaded onto it, with values between 0.429 and 0.737. This factor was labeled *“Academic Support*,* Learning Resources*,* Research Opportunities*,* and Facilities”*.
3. Factor 3 accounted for 2.98% of the variance, with an eigenvalue of 1.37. Eleven items loaded onto it, with values ranging from 0.498 to 0.718. This factor was labeled *“Program Impact on Professional Development and Essential Competencies”*.
4. Factor 4 explained 2.55% of the variance, with an eigenvalue of 1.17. Six items loaded onto it, with values between 0.547 and 0.749. This factor was labeled *“Quality*,* Accessibility*,* and Effectiveness of Learning Resources”*.

To increase reproducibility, per-factor internal consistency, inter-factor correlations, and item communalities were calculated. Internal consistency was good to excellent for all four factors: Cronbach’s alpha was 0.94 for Factor 1 (Program Learning Outcomes, Content, Educational Strategies, and Student Assessment), 0.94 for Factor 2 (Academic Support, Learning Resources, Research Opportunities, and Facilities), 0.95 for Factor 3 (Program Impact on Professional Development and Essential Competencies), and 0.90 for Factor 4 (Quality, Accessibility, and Effectiveness of Learning Resources). The four factors were substantially and significantly inter-correlated (Pearson’s *r* = 0.751–0.856, all *p* < 0.001; Factor 1–Factor 2: *r* = 0.856; Factor 1–Factor 3: *r* = 0.825; Factor 1–Factor 4: *r* = 0.795; Factor 2–Factor 3: *r* = 0.820; Factor 2–Factor 4: *r* = 0.800; Factor 3–Factor 4: *r* = 0.751), consistent with a coherent overarching satisfaction construct underlying the four-factor structure. Item communalities extracted from the four-factor solution ranged from 0.439 to 0.711 across the full 45-item set: 0.466–0.656 for Factor 1 items, 0.439–0.694 for Factor 2 items, 0.617–0.711 for Factor 3 items, and 0.635–0.687 for Factor 4 items.

These four factors define distinct aspects of the survey, confirming its construct validity. The survey was originally developed around five conceptual domains (program goals, curriculum content, learning resources, faculty support, and overall experience); however, the empirically derived EFA solution yielded four factors. This discrepancy arises because EFA is a data-driven technique that reflects the actual inter-item correlation structure rather than the a priori theoretical domains. In this case, items initially categorized under “academic support” and “learning resources” merged into a single EFA factor (Factor 2), suggesting that students perceived these two dimensions as closely related in their experience of the program. The four-factor solution is thus an empirically supported refinement of the original five-domain framework. Additionally, it should be noted that EFA was conducted on the full dataset rather than a randomly split half-sample, and no confirmatory factor analysis (CFA) was performed; this is acknowledged as a limitation, and CFA is recommended in future studies.

### Section 2: analysis of survey responses

#### Quantitative analysis of the survey

The study included a balanced representation of male (47.8%) and female (52.2%) graduates, with 92.3% being Egyptian. Most participants had high academic performance, with 33.5% obtaining an A and 23.2% an A+. The majority (75.3%) attended governmental Arabic schools, while only 8.1% were from high schools abroad (Arab countries). Regarding English proficiency, over half of the participants (51.7%) rated their proficiency as “Very Good”, suggesting a strong ability to engage with English-based medical content. Additionally, 53.8% lived with their families, while 28.8% resided in private accommodation, and the remaining used to reside in the university residence (Table 2).

**Table 2:** Demographic characteristics of the study participants:

Characteristic	*N*	%
Gender		
Male	3469	47.8%
Female	3795	52.2%
Nationality		
Egyptian	6703	92.3%
Non-Egyptian	561	7.7%
Last Obtained Grade (Graduation Grade)		
A+	1682	23.2%
A	2432	33.5%
B+	1612	22.2%
B	823	11.3%
C+	395	5.4%
C	204	2.8%
D+	37	0.5%
D	54	0.7%
F *(failed the regular final exams and passed the re-sit)*	25	0.3%
School Type		
Governmental (Arabic Schools)	5469	75.3%
Governmental (Language Schools)	307	4.3%
Private (Arabic Schools)	409	5.6%
Private (Language Schools)	199	2.7%
International (American Diploma, IG, IB)	277	3.8%
High School Attended Abroad (Arab Countries)	586	8.1%
High School Attended Abroad (Non-Arab Countries)	17	0.2%
English Language Proficiency		
Excellent	1933	26.7%
Very Good	3756	51.7%
Good	1441	19.8%
Fair	134	1.8%
Residence During College Time		
With Family	3907	53.8%
University Residence	1265	17.4%
Private Residence	2092	28.8%
Total	7264	100%

Table 3 shows that graduates reported moderate satisfaction across different program dimensions, with mean scores ranging from 2.71 to 3.41. Strengths included integration of basic and clinical sciences (3.25), availability of learning opportunities (3.33), and faculty consultation (3.41). However, the learning environment (2.71) and preparation for studying in the program (2.77) were rated lower, indicating challenges in student engagement and transition to the new curriculum. Assessment methods (2.91) and research opportunities (2.93) were also areas needing improvement, highlighting the need for better alignment between evaluation strategies and learning objectives.

**Table 3:** Descriptive statistics of the survey items and subscales

Item No.	Item Text	Mean (± SD)	Min – Max
Program Learning Outcomes, Content, Educational Strategies, and Student Assessment		3.08 (± 0.81)	
1	The program’s mission and goals were clear, appropriate, publicized, and reflected my aspirations to study in the program and obtain the degree	3.14 (± 1.04)	1–5
2	The learning outcomes of the program were generally clear	3.22 (± 1.02)	1–5
3	The learning outcomes of the different courses were clear	3.21 (± 1.00)	1–5
4	The program achieved the principle of integration between the basic sciences and each other, and between them and the clinical sciences	3.25 (± 1.07)	1–5
5	Program content was suitable for the requirements of the future doctor	3.06 (± 1.09)	1–5
6	I was effectively prepared to study in the program as it was a new program	2.77 (± 1.15)	1–5
7	The learning environment in the program was stimulating and encouraging	2.71 (± 1.10)	1–5
8	The program contains sufficient and appropriate elective courses to prepare graduates for various fields of specialization	3.12 (± 1.08)	1–5
9	The program offered enough learning opportunities (i.e., classes, lectures, clinical sessions, community-based activities …)	3.33 (± 1.07)	1–5
10	Teaching methods were variable and appropriate for the learning outcomes	3.06 (± 1.07)	1–5
11	The instructors used interactive teaching/learning methods (e.g., small group activities, discussion forums …)	3.23 (± 1.07)	1–5
12	Assessment methods were enough for measuring the learning outcomes	2.91 (± 1.10)	1–5
13	Teachers used the agreed-upon assessment methods as mentioned in each course specification	3.21 (± 1.04)	1–5
14	Assessment methods were fair	2.91 (± 1.10)	1–5
Academic Support and Facilities		3.01 (± 0.81)	
15	The instructors provided me with constructive feedback on strengths and areas for improvement whenever possible	2.94 (± 1.08)	1–5
16	The program offered adequate opportunities to participate in research projects	2.93 (± 1.12)	1–5
22	The learning opportunities provided by the program helped me develop my research skills	2.92 (± 1.11)	1–5
24	Physical facilities (i.e., lecture halls, laboratories, small group rooms …) were adequate and suitable	3.21 (± 1.14)	1–5
25	The student computer labs were sufficient for my needs	3.11 (± 1.13)	1–5
26	Adequate facilities were available for extracurricular activities (including sporting and recreational activities)	2.79 (± 1.11)	1–5
27	Field training activities (clinical training, practicum, cooperative training) were effective in developing my clinical skills	3.02 (± 1.11)	1–5
28	I was made aware of the academic support services provided (e.g., academic advising, specialized services for disabled students, study skills workshops …)	3.01 (± 1.11)	1–5
29	The program provided me with counseling and academic support whenever I needed it	2.93 (± 1.09)	1–5
30	The instructors were available for consultation and advice	3.41 (± 0.99)	1–5
31	The teachers were supportive and responsive to my needs and encouraged me to do my best	3.21 (± 1.03)	1–5
32	My academic progress was continuously monitored by my teachers who provided me with feedback on my achievement	2.73 (± 1.11)	1–5
33	The program faculty had significant knowledge of the program content, and this applies to technicians in laboratories, clinics, and training settings	2.90 (± 1.13)	1–5
34	Academic support services provided by the program were generally satisfactory	2.97 (± 1.07)	1–5
Program Impact on Professional Development and Essential Competencies		3.17 (± 0.86)	
35	The program goals inspired me to pursue a career in medicine	3.05 (± 1.12)	1–5
36	The program helped me develop the knowledge and skills I need to be a successful doctor	3.09 (± 1.06)	1–5
37	The program helped me to keep up to date with the latest developments in medicine	2.99 (± 1.08)	1–5
38	The program helped me develop the ability to investigate and solve new problems	3.13 (± 1.06)	1–5
39	The program helped me develop self-directed learning skills	3.37 (± 1.05)	1–5
40	The program helped me develop the ability to work effectively in teams	3.24 (± 1.05)	1–5
41	The program improved my communication and interpersonal skills	3.28 (± 1.06)	1–5
42	The program helped me develop critical thinking skills	3.19 (± 1.05)	1–5
43	The program helped me develop professionalism and commitment to ethical practice	3.38 (± 1.03)	1–5
44	The program helped me develop skills in using technology in learning	3.25 (± 1.08)	1–5
45	The program helped me develop and improve my abilities in the field of innovation and skill acquisition	2.94 (± 1.11)	1–5
Quality, Accessibility, and Effectiveness of Learning Resources		3.08 (± 0.87)	
17	The learning resources (e.g., textbooks, guides, web-based sources …) provided by the program were adequate to meet my needs	2.95 (± 1.13)	1–5
18	The learning resources provided by the program were up to date and accurate	3.06 (± 1.06)	1–5
19	The learning resources provided by the program were accessible and easy to use	3.27 (± 1.04)	1–5
20	The learning resources provided by the program supported students’ different learning styles (visual, auditory, kinesthetic)	3.10 (± 1.05)	1–5
21	The learning resources provided by the program helped me to become an independent learner	3.10 (± 1.09)	1–5
23	The learning resources provided by the program helped me prepare for assessments	2.99 (± 1.07)	1–5
Overall:			
Overall, I was satisfied with my educational experience in the program		2.94 (± 1.17)	1–5

Table 4 shows that statistically significant differences in overall satisfaction were observed across several student subgroups; however, all effect sizes were negligible to small, indicating that these differences, while statistically detectable in a large sample, are of limited practical significance. Male students (2.98) reported slightly higher satisfaction than females (2.90) (*p* = 0.006, Cohen’s *d* = 0.07). Higher-performing students (A+, 3.07) were more satisfied than lower-achieving students (D, 2.43) (*p* < 0.001, η² = 0.008). Students from private Arabic schools (2.99) were more satisfied than those from international schools (2.64) (*p* = 0.015, η² = 0.003). English proficiency also influenced satisfaction, with higher proficiency levels showing greater satisfaction (*p* < 0.001, η² = 0.004). Students residing in university housing (2.99) reported higher satisfaction than those in private residences (2.93) and those living with family (2.83) (*p* < 0.001, η² = 0.003). No statistically significant differences in overall satisfaction were detected between Egyptians and non-Egyptians (*p* = 0.077, Cohen’s *d* = 0.08). Every subgroup comparison yields a negligible effect size: Cohen’s d ranged from 0.07 to 0.08, well below the threshold conventionally considered small (d ≈ 0.2), and η² ranged from 0.003 to 0.008, below the threshold for even a small effect (η² ≈ 0.01). Although several of these differences reached statistical significance, this reflects the statistical power afforded by the very large sample rather than any practically or educationally meaningful disparity in graduates’ experience of the program across sex, academic performance, school type, English proficiency, residence, or nationality.

**Table 4:** Comparison of overall educational experience in the program among different groups of study participants

Characteristic	Mean (± SD)	*p*-value	Effect Size
Gender			
Male *(n= 3469)*	2.98 (± 1.20)	0.006*	Cohen’s *d* = 0.07 (negligible)
Female *(n= 3795)*	2.90 (± 1.13)		
Nationality			
Egyptian *(n= 6703)*	2.93 (± 1.16)	0.077	Cohen’s *d* = 0.08 (negligible)
Non-Egyptian *(n= 561)*	3.02 (± 1.24)		
Last Obtained Grade (Graduation Grade)			
A+ *(n= 1682)*	3.07 (± 1.17)	< 0.001*	η² = 0.008 (small)
A *(n= 2432)*	2.96 (± 1.11)		
B+ *(n= 1612)*	2.90 (± 1.20)		
B *(n= 823)*	2.87 (± 1.20)		
C+ *(n= 395)*	2.73 (± 1.21)		
C *(n= 204)*	2.71 (± 1.20)		
D+ *(n= 37)*	2.89 (± 1.31)		
D *(n= 54)*	2.43 (± 1.27)		
F *(n= 25)*	2.92 (± 1.58)		
School Type			
Governmental (Arabic Schools) *(n = 5469)*	2.90 (± 1.17)	0.015*	η² = 0.003 (negligible)
Governmental (Language Schools) *(n= 307)*	2.82 (± 1.12)		
Private (Arabic Schools) *(n= 409)*	2.99 (± 1.12)		
Private (Language Schools) *(n= 199)*	2.80 (± 1.19)		
International (American Diploma, IG, IB) *(n= 277)*	2.64 (± 1.28)		
High School Attended Abroad (Arab Countries) *(n= 586)*	2.88 (± 1.27)		
High School Attended Abroad (Non-Arab Countries) *(n= 17)*	3.14 (± 1.10)		
English Language Proficiency			
Excellent *(n= 1933)*	2.92 (± 1.24)	< 0.001*	η² = 0.004 (negligible)
Very Good *(n= 3756)*	2.93 (± 1.14)		
Good *(n= 1441)*	2.76 (± 1.15)		
Fair *(n= 134)*	2.69 (± 1.24)		
Residence During College Time			
With Family *(n= 3907)*	2.83 (± 1.18)	< 0.001*	η² = 0.003 (negligible)
University Residence *(n= 1265)*	2.99 (± 1.14)		
Private Residence *(n= 2092)*	2.93 (± 1.20)		

* Statistically significant

Overall satisfaction showed weak but statistically significant correlations with gender (-0.034, *p* = 0.004), academic performance (-0.082, *p* < 0.001), English proficiency (-0.048, *p* < 0.001), and residence (-0.043, *p* = 0.001). These findings suggest that male students, higher achievers, those with stronger English proficiency, and those in university housing reported slightly higher satisfaction levels. However, nationality and school type did not show significant correlations with satisfaction, indicating that these factors had a limited impact on students’ overall experience (Table 5).

**Table 5:** Correlation between overall satisfaction mean score and important independent variables

		Gender	Nationality	Last Obtained Grade	School Type	English Language Proficiency	Residence During College Time
Overall Satisfaction Mean Score	Spearman’s rho	-0.034	0.021	-0.082	-0.019	-0.048	0.043
	Sig. (2-tailed)	0.004*	0.074	< 0.001*	0.151	< 0.001*	0.001*
	N	7264	7264	7264	5916^§^	5916^§^	5916^§^

* Statistically significant

^§^Analyses for school type, English language proficiency, and residence are based on *N* = 5,916, as these items were not completed by all respondents.

#### Quantitative content analysis of the open-ended questions

Out of the study participants, 5947 responded to the open-ended questions *(n= 5947)*.

Table 6 shows that integration between subjects was the most appreciated aspect by the graduates (28.1%), followed by using diverse learning methods in the program (13.2%), early clinical exposure (12%) that allowed graduates to gain hands-on experience sooner, critical thinking focus (10.2%) that shifted learning from memorization to problem-solving, and the modular system (10.1%) that made learning structured and easier to follow. Other aspects (like the use of question banks and formative assessments and extended internship period) were also appreciated but to a lesser extent.

**Table 6:** Analysis of students’ responses to the open-ended question *“What did you like most about the program?”*

Question	Response Category/Key Points	Response Frequency and Percentage	Sample Quotes
What did you *like* most about the program?	Integration between subjects and learning basic sciences in a clinical context	1675 (28.1%)	*“The integration of basic and clinical subjects helped me connect the dots between theory and practice.”* *“Studying each system as a whole*,* including pathology*,* medicine*,* and surgery together*,* made it easier to retain information.”*
	Variety of learning methods (PBL, TBL, online learning)	782 (13.2%)	*“The program encouraged problem-solving rather than memorization, which helped me think critically.”*
	Early clinical exposure and practical training	716 (12%)	*“I liked the exposure to clinical training early on, which gave me hands-on experience.”*
	Focus on critical thinking and problem-solving	605 (10.2%)	*“I appreciate that we are encouraged to think critically and apply knowledge rather than just memorize facts.”*
	Structured learning through the modular system	598 (10.1%)	*“The modular system was beneficial because it grouped related subjects together instead of studying them separately.”*
	Hands-on experience with clinical cases	548 (9.2%)	*“The clinical rounds gave me real-life medical exposure that helped in understanding patient care.”*
	Development of research and communication skills	467 (7.9%)	*“The program helped improve my research skills and communication with patients and colleagues.”*
	Use of question banks and formative assessments	352 (5.9%)	*“Using question banks and formative assessments helped me prepare better for exams.”*
	Extended internship period for skill development	204 (3.4%)	*“The extended internship period allows for deeper hands-on experience before becoming a full doctor.”*

Table 7 exhibits the main complaints of the graduates based on their experience of the new program. The most reported complaints are the time constraints and the high academic load (38.4%). Other complaints were also reported – like lack of standardized textbooks and study materials, excessive exams, neglect of graduates’ mental health, and poor infrastructure – but by relatively smaller percentage of graduates.

**Table 7:** Analysis of students’ responses to the open-ended question *“What did you dislike most about the program?”*

Question	Response Category/Key Points	Response Frequency and Percentage	Sample Quotes
What did you *dislike* most about the program?	Time constraints and academic overload	2283 (38.4%)	*“The amount of content we had to study in a short time was overwhelming.”*
	Absence of standardized textbooks and study materials	641 (10.8%)	*“We didn’t have official textbooks, and each lecturer recommended different sources, which was confusing.”*
	Excessive exams in short periods with no review time	518 (8.7%)	*“Too many exams in a short time, with no chance to review properly.”*
	Some faculty members are not well trained for the new system	501 (8.4%)	*“Some faculty members were not familiar with the new system, making it harder for us to understand.”*
	Neglect of students’ mental health due to high pressure	465 (7.8%)	*“Mental health support was nonexistent, and the pressure was unbearable.”*
	Poor infrastructure and lack of resources	369 (6.2%)	*“Some universities lacked proper facilities, including internet access, study spaces, and well-equipped hospitals for training.”*

The graduates gave several important suggestions for improving the new program as shown in Table 8. Extending module durations was the most frequent suggestion (25%) indicating that there is redundancy in the content. Next was the suggestion of providing study guides (17%) to eliminate confusion and keep them focused on what needs to be learned. Other important suggestions included reducing the unneeded content in courses and focusing on relevant, clinically useful material (14.9%), planning more clinical training and patient exposure for hands-on practice (13.3%). In addition, some graduates gave other helpful suggestions, as shown in Table 8.

**Table 8:** Analysis of students’ responses to the open-ended question *“What are your suggestions for improving the program?”*

Question	Response Category/Key Points	Response Frequency and Percentage	Sample Quotes
What are your *suggestions* for improving the program?	Extend module durations to match workload	1487 (25%)	*“Extend the duration of some modules so we can actually understand the content.”*
	Provide standardized study guides	1012 (17%)	*“Provide official textbooks or study guides for each module instead of making us look for random resources.”*
	Reduce unnecessary content and focus on essentials	885 (14.9%)	*“Remove unnecessary topics and focus on clinically relevant information.”*
	Plan more clinical training to increase patient exposure	789 (13.3%)	*“Increase early clinical training opportunities to make us more prepared for real practice.”*
	Train faculty members on the new system	488 (8.2%)	*“Professors should receive proper training on how to teach under the new system.”*
	Allow more preparation time for exams, reduce pressure	457 (7.7%)	*“Reduce the number of exams or at least give us enough time to prepare.”*
	Better time management and learning activities scheduling	389 (6.5%)	*“Improve scheduling so that we don’t feel rushed all the time.”*
	Increase student support and mental health resources	344 (5.8%)	*“Provide mental health support services for students who are struggling with stress.”*

## Discussion

The adoption of Competency-Based Medical Education (CBME) by the Supreme Council of Egyptian Universities signifies a groundbreaking transition from the conventional time-based training model to an outcome-driven approach. This new framework prioritizes equipping medical graduates with the critical competencies necessary for effective medical practice. Aligning with global advancements in medical education, this shift underscores the value of integrating practical skills and competencies, which have been shown to enhance medical outcomes significantly [18].

In this nationwide cross-sectional study, we examined the new Egyptian undergraduate medical program from the perspectives of 7264 medical students from 27 medical colleges. This represents a national program evaluation of CBME implementation, in which graduates’ satisfaction functions as a Level 1 (Reaction) indicator within the broader evaluation framework rather than as the sole object of interest. The study examined their satisfaction levels, perceived strengths, challenges encountered, and recommendations for improvement. Bland et al. noted that students play a crucial role in supporting curricular changes, with their feedback and enthusiasm serving as vital elements in maintaining and advancing these changes [19].

Our participant demographics were broadly consistent with those reported in other medical education studies, such as An et al., however, representativeness remains limited by the self-selection sampling approach [20].

The quantitative data on students’ perspectives in this study indicated that most graduates reported satisfaction across several examined program dimensions. These dimensions included: (1) Program Learning Outcomes, Content, Educational Strategies, and Student Assessment, (2) Academic Support and Facilities, (3) Program Impact on Professional Development and Essential Competencies, (4) Quality, Accessibility, and Effectiveness of Learning Resources, and (5) Overall Satisfaction. The average scores for these dimensions ranged from 2.71 to 3.41, reflecting a generally moderate level of satisfaction with various aspects of the program.

Notable satisfaction with the first program dimension—comprising Program Learning Outcomes, Content, Educational Strategies, and Student Assessment—stemmed from the effective integration of basic and clinical sciences, the clarity of program and course learning outcomes, and the provision of diverse learning opportunities. These results align with Shetty et al. findings, who investigated student perceptions of different components of the CBME curriculum and found that approximately 70% of students agreed that aligned and integrated topics facilitated learning beyond individual subjects [21]. Furthermore, Thakuria et al. examined students’ knowledge, experiences, and perceptions of the newly introduced competency-based medical education (CBME) curriculum in an Indian institution, revealing that 51.7% of participants acknowledged the significance of vertical and horizontal integration in enhancing their understanding of various topics [22]. Nevertheless, it should be noted that even for this highest-rated dimension (mean 3.08), satisfaction remained only moderately above the scale midpoint, suggesting that whilst graduates recognized the program’s intentions in these areas, there remains meaningful room for improvement in the consistency of delivery and implementation across institutions.

Student satisfaction with the second examined program dimension, Academic Support and Facilities, was primarily influenced by their perceptions of faculty support, including the availability of faculty members for consultation, as well as the quality of facilities provided by the program. These findings indicate that students viewed these aspects positively, highlighting their crucial role in fostering an environment that supports effective learning and academic success.

For instance, research by Wisenthige et al., found that students’ satisfaction with academic support was strongly linked to faculty accessibility—and that this support, combined with lecturers’ broader knowledge and quality of delivery, was critical for academic success [23]. However, the dimension mean for Academic Support and Facilities (3.01) sits barely above the scale midpoint and should not be read as strong endorsement. Moderate ratings of this kind indicate that approximately as many graduates were dissatisfied as satisfied with support provision, and institutions should treat these scores as a signal that targeted, sustained investment in faculty accessibility and facility quality remains necessary. Notably, faculty consultation was the single highest-rated individual item across the entire survey, which may reflect the close mentorship culture and small-group clinical teaching characteristic of Egyptian medical schools, where students have direct, personalized access to faculty during bedside and small-group sessions even where broader institutional support infrastructure lags behind.

Students’ opinions on the program’s impact on professional development, particularly regarding essential competencies, indicated that the majority recognized its role in fostering professionalism and a strong commitment to ethical practice. Within this third examined program dimension, self-directed learning skills, as well as communication and interpersonal abilities, were also highly valued. These findings align with Shetty et al., who reported that three-fourths of their students emphasized the importance of integrating attitude, ethics, and communication into the program. Their study highlighted how this approach not only enhanced students’ awareness of communication’s significance in healthcare but also underscored the essential role of attitude, ethics, and communication in doctor-patient relationships [21]. Similarly, Chakraborty et al. found consistent results [24].

Furthermore, Patil et al. examined undergraduate medical students’ perspectives on CBME and the associated challenges. Their study revealed that two-thirds of respondents recognized self-directed learning (SDL) sessions as crucial for fostering self-motivation and enhancing creativity [25]. However, with the mean for this dimension at 3.17, caution is warranted: while this is the highest-rated dimension, a mean that only modestly exceeds the midpoint reflects a neutral-to-moderate perception rather than confident endorsement. Educators should seek to understand why a substantial minority of graduates did not rate these competency outcomes more favorably, and whether structural features of the program are limiting the realization of its professional development goals.

Regarding the fourth examined program dimension—quality, accessibility, and effectiveness of learning resources—students expressed satisfaction with the availability, accessibility, and support provided by these resources. Their positive feedback suggests that institutions have effectively addressed students’ academic needs by ensuring that learning materials are both comprehensive and user-friendly.

These findings align with Sharma et al., who reported that a majority of students found digital learning resources highly accessible and instrumental in enhancing their academic performance [26]. That said, satisfaction with learning resources, whilst moderate (mean 3.08), should be interpreted carefully. Means clustering near the midpoint indicate that a considerable proportion of graduates were not satisfied with resource quality and accessibility, a balance of opinion that warrants continued attention from program administrators rather than complacency.

The fifth examined program dimension was overall satisfaction levels. The overall mean satisfaction score of approximately 2.94 falls marginally below the midpoint of the 5-point scale (3.0), and this finding must be interpreted with care. An overall mean below the midpoint indicates that, on average, graduates were slightly below neutral in their satisfaction with the program as a whole; a result that cannot and should not be characterized as a positive endorsement of the reform. This does not negate the program’s genuine strengths, but it does signal that the reformed curriculum has not yet achieved majority graduate endorsement, and that complacency in its further development would be unwarranted. Our study revealed that male students, high achievers, those with strong English proficiency, and students living in university housing reported slightly higher satisfaction. These findings resonate with prior research. For instance, Li et al. found that male students and high achievers tend to report greater satisfaction with their educational experiences [27]. However, it is important to note that while these subgroup differences reached statistical significance (as expected given the large sample size (*N* = 7,264) all correlation coefficients were small (Spearman’s rho range: −0.034 to − 0.082, Table 5), indicating that these variables account for a limited proportion of the variance in satisfaction scores. These findings should therefore not be over-interpreted as evidence of practically meaningful differences between subgroups.

The higher satisfaction observed among high achievers and students with stronger English proficiency can be attributed to several factors. High achievers typically have a solid understanding of the material, which boosts their confidence and ability to navigate the program effectively, ultimately leading to a more positive evaluation of the program [28].

Additionally, English proficiency plays a critical role in medical education, where English is commonly used as the primary medium of instruction for technical terms and research. Students with strong English skills are better equipped to comprehend lectures, textbooks, and exams, minimizing frustration and enriching their learning experience [29]. Furthermore, proficiency in English enables active participation in discussions, presentations, and collaborative projects, fostering engagement and satisfaction within the academic environment [30].

Although students demonstrated moderate satisfaction across almost all of the survey items, the results also highlighted a few challenges they encountered regarding their preparedness for studying in the program. These challenges were reflected in lower ratings (2.71–2.77), highlighting obstacles in their engagement and ability to adapt to the new curriculum. This observation is supported by prior research, which shows that transitioning to CBME often requires additional guidance and support mechanisms, particularly for students accustomed to traditional, structured learning methods. Such research emphasizes the value of an initial adaptation phase that could be enhanced by improved preparatory or support systems [31]. Although the national curriculum implemented in all our medical colleges includes an orientation period, the low student ratings, in this study suggest that the duration is insufficient and should be extended for better effectiveness.

The three lowest-rated individual survey items; which are: learning environment (2.71), student preparedness for the program (2.77), and extracurricular facilities (2.79); fall below the scale midpoint, indicating net dissatisfaction with these specific aspects. A plausible explanation for the learning environment item scoring lowest is that the curriculum reform raised students’ expectations regarding the quality of the learning environment. Exposure to student-centered and competency-based educational approaches may have increased expectations for flexible learning spaces, access to educational technologies, and supportive learning climates. Consequently, students may have rated the learning environment more critically, even if objective improvements had occurred. These ratings carry direct practical implications. The low score for learning environment suggests that physical and psychosocial conditions for learning require structural attention, particularly given that students identified mental health pressures and infrastructure deficits in their open-ended responses. The low preparedness score indicates that transition support mechanisms (including orientation programs and induction activities) are insufficient for the demands of the reformed curriculum, and should be substantially extended and redesigned. The low score for extracurricular facilities points to a need for investment in student welfare infrastructure beyond the classroom.

Moreover, students identified assessments as an area in need of improvement, expressing concerns about the excessive number of exams. This issue may stem from their familiarity with traditional assessment methods, which primarily consist of a limited range of evaluations. These conventional approaches are insufficient for Competency-Based Medical Education (CBME), which prioritizes the development of practical skills, ethical reasoning, and critical thinking competencies that cannot be effectively measured through a limited number or variety of exams. Notably, the assessment dimension mean was moderate rather than markedly low, suggesting that while the sheer frequency of examinations was a source of dissatisfaction, students did not uniformly reject the diversified assessment formats themselves; some may have viewed methods such as OSCEs and portfolios as more authentic or clinically relevant than the single high-stakes exams typical of the former program, producing a mixed rather than uniformly negative evaluation.

Research underscores the importance of aligning assessments with curriculum objectives and incorporating diverse evaluation methods, providing meaningful feedback, identifies learning gaps, and ensures that students acquire the necessary competencies for professional practice [32].

To ensure a well-balanced assessment strategy, the nationally implemented new program incorporates a variety of evaluation methods, including formative assessments, objective structured clinical examinations (OSCEs), portfolios, modified essay questions, multiple-choice questions (MCQs), and performance-based tasks. However, this diverse assessment approach may contribute to students’ perception that the number of exams is excessive. Enhancing transparency in assessment design, providing clear guidelines, and offering structured support may help students better understand the purpose and expectations of these diverse evaluation methods, ultimately improving their assessment experience.

The quantitative content analysis data derived from students’ open-ended responses revealed several key aspects of the program that were highly valued. These aspects include subject integration, diverse learning methodologies, early clinical exposure (ECE) which enabled hands-on experience, and a focus on critical thinking that shifted learning from rote memorization to problem-solving. These perspectives align with numerous studies highlighting the positive reception of integration in competency-based medical education (CBME). A study conducted on students’ perspectives regarding the CBME curriculum at a school in India revealed that the importance of integration was widely acknowledged across various cohorts, with more recent groups, such as those from 2022 to 2023 giving higher ratings to this aspect [26]. Patil et al. reported that most of their students acknowledged problem-based learning (PBL) as a tool for enhancing critical thinking and decision-making skills. Additionally, they agreed that vertical and horizontal integration facilitated a better understanding of topics. They also recognized that Early Clinical Exposure is valuable in strengthening their comprehension of fundamental scientific concepts and their application to clinical specialties [25]. One study found that ECE was widely recognized as an effective and valuable strategy, with 89.2% of students expressing favorable views toward it [33]. Similarly, other researchers confirmed the benefits of ECE as an educational approach, reporting comparable positive perspectives [34].

In addition to the program’s strengths, students also identified certain constraints in their open-ended responses. Notably, 38.4% reported issues related to time constraints and academic overload. This may be attributed to the nature of Competency-Based Medical Education (CBME), which prioritizes mastery of specific skills and competencies. While CBME offers the advantage of personalized, self-paced learning coupled with rigorous assessments, it can also impose significant pressure on students to meet strict deadlines amid demanding workloads [35]. Additionally, the recent introduction of interdisciplinary integration in many Egyptian colleges may have led to redundancies and overlapping content between disciplines, further contributing to content overload.

Based on the study findings and students’ suggestions, some actionable recommendations emerge to enhance the implementation of the new implemented Egyptian curriculum. This includes extending the orientation period to ease the transition from traditional curricula, regular reviews of course content to reduce redundancy. Orientation to the students regarding the importance of the frequent assessment and in the meantime reducing the frequency of assessments to some extent to alleviate academic strain, and to help students manage increased pressures. Continuous professional development for faculty is also crucial to strengthening their ability to effectively implement the new program. Collectively, these recommendations address key challenges and aim to maximize the program’s effectiveness and overall impact.

This study has some limitations that should be acknowledged. First, although the survey included over 7,000 students, respondents represented 40.7% of the total population, which raises the possibility of non‑response bias. Unfortunately, detailed data on non‑respondents were not available, limiting our ability to compare their characteristics with those of respondents. Second, the use of convenience sampling may limit generalizability, and the reliance on self-reported data introduces the potential for social desirability bias—both of which are well-recognized limitations in cross-sectional survey designs. Despite these limitations, the large sample size and alignment of participant demographics with previous studies support the robustness of our conclusions. Third, the EFA was conducted on the full dataset rather than a randomly split half-sample, and no confirmatory factor analysis (CFA) was performed; future studies should employ a split-sample design and CFA to cross-validate the factor structure. Fourth, the very high Cronbach’s alpha (0.98) may reflect item redundancy in addition to strong internal consistency; future instrument revisions should examine item-total correlations to identify and remove redundant items. Fifth, consistent with the Kirkpatrick framework adopted in this study, our findings correspond exclusively to Level 1 (Reaction): they capture graduates’ perceptions of and satisfaction with the reformed program, rather than demonstrated learning gains (Level 2), changes in clinical behaviour (Level 3), or downstream patient or health-system outcomes (Level 4). Future research employing objective performance measures and longitudinal designs is needed to evaluate these higher-order levels. Sixth, inter-rater reliability for the quantitative content analysis of open-ended responses was not formally quantified, as independent pre-consensus codes were not retained; future studies should calculate and report a formal agreement statistic (e.g., Cohen’s κ) before consensus discussion. Finally, all statistically significant subgroup differences in satisfaction were of small effect size (Spearman’s rho: −0.034 to − 0.082) and should be interpreted with caution, as they may not represent practically meaningful differences.

## Conclusion

In conclusion, from the students’ perspective, implementing the Competency-Based Medical Education (CBME) framework in Egypt marks a significant transition from traditional training to a competency-focused approach designed to better prepare graduates for effective practice. This shift has introduced notable strengths, including subject integration, diverse learning opportunities, enhanced professionalism, self-directed learning, early clinical exposure, and improved critical thinking. However, students have also faced challenges, particularly in adapting to the new environment, managing assessments, and coping with increased academic workloads. Addressing these issues is essential for optimizing the overall effectiveness of the CBME framework.

The practical implications of these findings are specific and actionable. First, the below-midpoint overall satisfaction mean (≈ 2.94) signals that the program has not yet achieved the level of graduate endorsement that would justify complacency in its implementation. Second, the three lowest-rated items (learning environment: 2.71, student preparedness: 2.77, and extracurricular facilities: 2.79) each falling below the scale midpoint, identify concrete priority areas for institutional action: redesigning and extending orientation programs, improving the physical and psychosocial learning environment, and expanding student welfare and recreational infrastructure. Third, the consistently expressed concerns about workload and assessment frequency across both quantitative and open-ended responses indicate a need to review module scheduling, content load, and the sequencing of assessments to reduce cognitive overload without compromising the competency-based model. Fourth, the gap between students with strong and weak English proficiency, whilst small in effect size, points to the value of targeted English language support, particularly for students from governmental Arabic schools who constitute the majority of graduates. Addressing these priorities systematically, rather than incrementally, is essential if the reformed Egyptian medical program is to achieve the level of graduate endorsement commensurate with its ambitions.

### Future directions

This study identifies key priorities for future research to evaluate and sustain the impact of undergraduate medical education reform in Egypt. Future studies should examine employers’ and hospital directors’ perceptions of graduates from the CBME (5 + 2) system. Longitudinal research is needed to correlate medical interns’ performance during the extended two-year internship with their foundational five-year undergraduate training, monitor satisfaction across successive cohorts, and compare outcomes with graduates of the former 6 + 1 program to assess the reform’s long-term effectiveness. Collectively, these efforts will provide a more comprehensive understanding of the reform and inform ongoing improvements in medical education in Egypt and beyond.

## Supplementary Information

Supplementary Material 1.

## Data Availability

Data and materials are available by contacting the corresponding author.

## Abbreviations

CBME: Competency-Based Medical Education
ECE: Early Clinical Exposure
EFA: Exploratory Factor Analysis
IRB: Institutional Review Board
KMO: Kaiser-Meyer-Olkin
MCQs: Multiple Choice Questions
NARS-Medicine: National Academic Reference Standards for Medicine
OSCEs: Objective Structured Clinical Examinations
PBL: Problem-Based Learning
RUMP: Reform of Undergraduate Medical Program
SCU: Supreme Council of Egyptian Universities
SDL: Self-Directed Learning
SPSS: Statistical Package for Social Sciences
UME: Undergraduate Medical Education
