# Supplementary material for: Evaluation of the new Egyptian undergraduate medical program from students’ perspectives: a nationwide cross-sectional study
Source: BMC Med Educ. 2026 Sep 8;26:1446. doi: 10.1186/s12909-026-10200-2 (PMC13555892; doi:10.1186/s12909-026-10200-2)
Supplement: Supplementary file 1 [file 12909_2026_10200_MOESM1_ESM.docx]

New 5+2 Medicine Program Evaluation

*Final Year Medical Students’ Survey*

Name of Medical School & University: ………………………

Type of Medical School: [	] Governmental	[	] Public	[	] Private	[	] Military

*Dear Medical Students of the 5+2 Program,*

The purpose of this survey is to evaluate the “5+2 Bachelor of Medicine and Surgery Program” from your point of view. Your feedback is important in this regard, and it will be used to improve the program for future students. The survey is anonymous and confidential. Please choose the answer that is closest to your personal experience as much as possible.

Thank you for your time and effort.

Part 1: Demographic Data:

**Name *(optional)*:** ………………………….	**Age:** ………………………….

Gender: [	] Male	[	] Female	Year of Graduation: ………………………….

Nationality:	[	] Egyptian	[	] Non-Egyptian

Last Obtained Grade:	[	] A+	[	] A	[	] B+	[	] B	[	] C+	[	] C	[	] D+	[	] D	[	] F

High School Type:	[	] Governmental (Arabic Schools)	[	] Governmental (Language Schools)	[	] Private (Arabic Schools)	[	] Private (Language Schools)	[	] International (American Diploma, IG, IB)	[	] High School Attended Abroad (Arab Countries)	[	] High School Attended Abroad (Non-Arab Countries)

English Language Proficiency: [	] Excellent	[	] Very Good	[	] Good	[	] Fair

Residence During College Time: [	] With Family	[	] University Residence	[	] Private

Part 2:

Direction: For each of the statements in the table below, choose the option that represents your opinion, where:

- Strongly Agree (SA): You are in full agreement with the statement and believe it to be totally true.
- Agree (A): You agree with the statement and believe it to be mostly true.
- Sometimes (S): You believe the statement to be true some of the time, but not always.
- Disagree (D): You do not agree with the statement and believe it to be mostly false.
- Strongly Disagree (SD): You completely oppose the statement and believe it to be totally false.
- Not Applicable (NA): The statement does not apply to your case.

| **No.** | **Statement** | **SA** | **A** | **S** | **D** | **SD** | **NA** |
| --- | --- | --- | --- | --- | --- | --- | --- |
| **Section 1: Program Objectives, Content, and Learning Opportunities:** | | | | | | | |
| 1 | The program's mission and goals were clear, appropriate, publicized, and reflecting my aspirations to study in the program and obtain the degree |  |  |  |  |  |  |
| 2 | The learning outcomes (ILOs) of the program as a whole were clear |  |  |  |  |  |  |
| 3 | The Learning Outcomes (ILOs) of the different courses were clear |  |  |  |  |  |  |
| 4 | The program achieved the principle of integration between the basic sciences and each other, and between them and the clinical sciences |  |  |  |  |  |  |
| 5 | Program content was suitable for the requirements of the future doctor |  |  |  |  |  |  |
| 6 | I was effectively prepared to study in the program as it was a new program |  |  |  |  |  |  |
| 7 | The learning environment in the program was stimulating and encouraging |  |  |  |  |  |  |
| 8 | The program contains sufficient and appropriate elective courses to prepare graduates for various fields of specialization |  |  |  |  |  |  |
| 9 | The program offered enough learning opportunities (i.e., classes, lectures, clinical sessions, community-based activities …) |  |  |  |  |  |  |
| 10 | Teaching methods were variable and appropriate for the learning outcomes |  |  |  |  |  |  |
| 11 | The instructors used interactive teaching/learning methods (e.g., small group activities, discussion forums …) |  |  |  |  |  |  |
| 12 | Assessment methods were enough for measuring the learning outcomes |  |  |  |  |  |  |
| 13 | Teachers used the agreed-upon assessment methods as mentioned in each course specification |  |  |  |  |  |  |
| 14 | Assessment methods were fair |  |  |  |  |  |  |
| 15 | The instructors provided me with constructive feedback on strengths and areas for improvement whenever possible |  |  |  |  |  |  |
| 16 | The program offered adequate opportunities to participate in research projects |  |  |  |  |  |  |
| **If you have additional comments related to this section of the questionnaire, please add them here:**  …………………………………………………………………………………………………………………………………………………  …………………………………………………………………………………………………………………………………………………  ………………………………………………………………………………………………………………………………………………… | | | | | | | |
| **Section 2: Learning Resources:** | | | | | | | |
| 17 | The learning resources (e.g., textbooks, guides, web-based sources …) provided by the program were adequate to meet my needs |  |  |  |  |  |  |
| 18 | The learning resources provided by the program were up-to-date and accurate |  |  |  |  |  |  |
| 19 | The learning resources provided by the program were accessible and easy to use |  |  |  |  |  |  |
| 20 | The learning resources provided by the program supported students’ different learning styles (visual, auditory, kinesthetic) |  |  |  |  |  |  |
| 21 | The learning resources provided by the program helped me to learn independently |  |  |  |  |  |  |
| 22 | The learning resources provided by the program helped me develop my research skills |  |  |  |  |  |  |
| 23 | The learning resources provided by the program helped me to prepare for assessments |  |  |  |  |  |  |
| 24 | Physical facilities (i.e., lecture halls, laboratories, small group rooms …) were adequate and suitable |  |  |  |  |  |  |
| 25 | The student computer labs were sufficient for my needs |  |  |  |  |  |  |
| 26 | Adequate facilities were available for extracurricular activities (including sporting and recreational activities) |  |  |  |  |  |  |
| 27 | Field training activities (clinical training, practicum, cooperative training) were effective in developing my clinical skills |  |  |  |  |  |  |
| **If you have additional comments related to this section of the questionnaire, please add them here:**  …………………………………………………………………………………………………………………………………………………  …………………………………………………………………………………………………………………………………………………  ………………………………………………………………………………………………………………………………………………… | | | | | | | |
| **Section 3: Academic Guidance and Support:** | | | | | | | |
| 28 | I was made aware of the academic support services provided (e.g., academic advising, specialized services for disabled students, study skills workshops …) |  |  |  |  |  |  |
| 29 | The program provided me with counseling and academic support whenever I needed it |  |  |  |  |  |  |
| 30 | The instructors were available for consultation and advice |  |  |  |  |  |  |
| 31 | The teachers were supportive and responsive to my needs and encouraged me to do my best |  |  |  |  |  |  |
| 32 | My academic progress was continuously monitored by my teachers who provided me with feedback on my achievement |  |  |  |  |  |  |
| 33 | The program faculty had significant knowledge of the program content, and this applies to technicians in laboratories, clinics, and training settings |  |  |  |  |  |  |
| 34 | Overall, academic support services provided by the program were satisfactory |  |  |  |  |  |  |
| **If you have additional comments related to this section of the questionnaire, please add them here:**  …………………………………………………………………………………………………………………………………………………  …………………………………………………………………………………………………………………………………………………  ………………………………………………………………………………………………………………………………………………… | | | | | | | |
| **Section 4: Program Impact on Students:** | | | | | | | |
| 35 | The program inspired me to pursue a career in medicine |  |  |  |  |  |  |
| 36 | The program helped me develop the knowledge and skills I need to be a successful doctor |  |  |  |  |  |  |
| 37 | The program helped me to keep up-to-date with the latest developments in medicine |  |  |  |  |  |  |
| 38 | The program helped me develop the ability to investigate and solve new problems |  |  |  |  |  |  |
| 39 | The program helped me develop self-directed learning skills |  |  |  |  |  |  |
| 40 | The program helped me develop the ability to work effectively in teams |  |  |  |  |  |  |
| 41 | The program improved my communication and interpersonal skills |  |  |  |  |  |  |
| 42 | The program helped me develop critical thinking skills |  |  |  |  |  |  |
| 43 | The program helped me develop professionalism and commitment to ethical practice |  |  |  |  |  |  |
| 44 | The program helped me develop skills in using technology in learning |  |  |  |  |  |  |
| 45 | The program helped me develop and improve my abilities in the field of innovation and scientific research |  |  |  |  |  |  |
| **If you have additional comments related to this section of the questionnaire, please add them here:**  …………………………………………………………………………………………………………………………………………………  …………………………………………………………………………………………………………………………………………………  ………………………………………………………………………………………………………………………………………………… | | | | | | | |
| **Section 5: Overall Satisfaction:** | | | | | | | |
| 46 | Overall, I was satisfied with my educational experience in the program |  |  |  |  |  |  |
| 47 | I feel ready to graduate and obtain a license to practice the profession |  |  |  |  |  |  |
| **If you have additional comments related to this section of the questionnaire, please add them here:**  …………………………………………………………………………………………………………………………………………………  …………………………………………………………………………………………………………………………………………………  ………………………………………………………………………………………………………………………………………………… | | | | | | | |
| **Open-ended Questions:** | | | | | | | |
| - **What did you like most about the program?**   …………………………………………………………………………………………………………………………………………………………………………………………………………………………………………………………………………………………… | | | | | | | |
| - **What did you dislike most about the program?**   ……………………………………………………………………………………………………………………………………………………………………………………………………………………………………………………………………………………………… | | | | | | | |
| - **What suggestions do you have for improving the program?**   ……………………………………………………………………………………………………………………………………………………………………………………………………………………………………………………………………………………………… | | | | | | | |

*Thank you …*
